# Socioeconomic position and consumption of sugary drinks, sugar-sweetened beverages and 100% juice among Canadians: a cross-sectional analysis of the 2015 Canadian Community Health Survey–Nutrition

**DOI:** 10.17269/s41997-021-00602-8

**Published:** 2022-02-09

**Authors:** Christine Warren, Erin Hobin, Douglas G. Manuel, Laura N. Anderson, David Hammond, Mahsa Jessri, JoAnne Arcand, Mary L’Abbé, Ye Li, Laura C. Rosella, Heather Manson, Brendan T. Smith

**Affiliations:** 1 Public Health Ontario, 480 University Avenue, Suite 300, Toronto, ON M5G 1V2 Canada; 2 Department of Nutritional Sciences, University of Toronto, Toronto, ON Canada; 3 Dalla Lana School of Public Health, University of Toronto, Toronto, ON Canada; 4 Clinical Epidemiology, Ottawa Hospital Research Institute, Ottawa, ON Canada; 5 Institute for Clinical Evaluative Sciences – Central Site, Toronto, ON Canada; 6 Health Analysis Division, Statistics Canada, Ottawa, ON Canada; 7 Department of Family Medicine, and School of Epidemiology and Public Health, University of Ottawa, Ottawa, ON Canada; 8 Bruyère Research Institute, Ottawa, ON Canada; 9 Department of Health Research Methods, Evidence, and Impact, McMaster University, Hamilton, ON Canada; 10 Child Health Evaluative Sciences, SickKids Research Institute, Toronto, ON Canada; 11 School of Public Health and Health Systems, University of Waterloo, Waterloo, ON Canada; 12 Food, Nutrition and Health Program, University of British Columbia, Vancouver, BC Canada; 13 Faculty of Health Sciences, Ontario Tech University, Oshawa, ON Canada; 14 Institute for Better Health, Trillium Health Partners, Mississauga, ON Canada; 15 Vector Institute, Toronto, ON Canada; 16 Temerty Faculty of Medicine, Toronto, ON Canada

**Keywords:** 2015 Canadian Community Health Survey–Nutrition, Social inequities, Socioeconomic position, Sugar-containing beverages, Sugary drinks, Sugar-sweetened beverages, 100% juice, Enquête sur la santé dans les collectivités canadiennes – Nutrition 2015, iniquités sociales, statut socioéconomique, boissons contenant du sucre, boissons sucrées, boissons contenant du sucre ajouté, jus pur à 100 %

## Abstract

**Objective:**

The aim of this study was to describe sugary drink (beverages with free sugars), sugar-sweetened beverage (beverages with added sugars, SSB) and 100% juice (beverages with natural sugars) consumption across socioeconomic position (SEP) among Canadians.

**Methods:**

We conducted a cross-sectional analysis of 19,742 respondents of single-day 24-h dietary recalls in the nationally representative 2015 Canadian Community Health Survey–Nutrition. Poisson regressions were used to estimate the prevalence of consuming each beverage type on a given day. Among consumers on a given day, linear regressions were used to estimate mean energy intake. Models included household education, food security and income quintiles as separate unadjusted exposures. Sex-specific models were estimated separately for children/adolescents (2–18 years) and adults (19 +).

**Results:**

Among female children/adolescents, the prevalence of consuming sugary drinks and, separately, SSB ranged from 11 to 21 and 8 to 27 percentage-points higher among lower education compared to ‘Bachelor degree or above’ households. In female adults, the prevalence of consuming sugary drinks and, separately, SSB was 10 (95% CI: 1, 19) and 14 (95% CI: 2, 27) percentage-points higher in food insecure compared to secure households. In males, the prevalence of consuming 100% juice was 9 (95% CI: − 18, 0) percentage-points lower among food insecure compared to secure households. Social inequities in energy intake were observed in female adult consumers, among whom mean energy from sugary drinks was 27 kcal (95% CI: 3, 51) higher among food insecure compared to secure and 35 kcal (95% CI: 2, 67) higher from 100% juice among ‘less than high school’ education compared to ‘Bachelor degree or above’ households.

**Conclusion:**

Social inequities in sugary drink consumption exist in Canada. The associations differed by SEP indicator. Equitable interventions to reduce consumption are warranted.

## Résumé

**Objectif:**

Dresser le portrait de la consommation de boissons sucrées (boissons contenant des sucres libres), de boissons contenant du sucre ajouté et de jus purs à 100 % (boissons contenant des sucres naturels) chez la population canadienne en fonction du statut socioéconomique.

**Méthode:**

Nous avons effectué une analyse transversale des rappels alimentaires de 24 heures réalisés par 19 742 personnes dans le cadre de l’Enquête sur la santé dans les collectivités canadiennes – Nutrition 2015, laquelle est représentative de la population nationale. Nous avons utilisé la régression de Poisson pour estimer la prévalence de la consommation de chaque type de boisson durant un jour donné. Pour calculer l’apport énergétique quotidien moyen, nous avons utilisé des régressions linéaires. Les modèles d’analyse traitent la scolarité, la sécurité alimentaire et les quintiles de revenu des ménages comme des expositions non ajustées distinctes. L’analyse en fonction du sexe est divisée en deux groupes, soit les enfants et adolescents (2 à 18 ans) et les adultes (19 ans et plus).

**Résultats:**

Chez les filles et les adolescentes, la prévalence de la consommation de boissons sucrées et de boissons contenant du sucre ajouté est plus élevée (de 11 à 21 points de pourcentage et de 8 à 27 points de pourcentage, respectivement) dans les ménages moins scolarisés que dans les ménages plus scolarisés (baccalauréat et études supérieures). Chez les femmes vivant de l’insécurité alimentaire, la prévalence de la consommation de boissons sucrées et de boissons contenant du sucre ajouté est plus élevée de 10 points de pourcentage (IC de 95 % : 1, 19) et de 14 points de pourcentage (IC de 95 % : 2, 27), respectivement, que chez celles qui n’en vivent pas. Chez les sujets masculins, la prévalence de consommation des jus purs à 100 % est moins élevée de 9 points de pourcentage (IC de 95 % : -18, 0) dans les ménages vivant de l’insécurité alimentaire. Nous avons remarqué des disparités sur le plan social dans l’apport énergétique chez les femmes; l’apport moyen provenant de boissons sucrées est plus élevé de 27 kcal (IC de 95% : 3, 51) dans les ménages vivant de l’insécurité alimentaire, et l’apport moyen provenant de jus pur à 100 % est plus élevé de 35 kcal (IC de 95 % : 2, 67) dans les ménages moins scolarisés (pas de diplôme d’études secondaires) que chez les ménages plus scolarisés (baccalauréat et études supérieures).

**Conclusion:**

Il existe des disparités sur le plan social dans la consommation de boissons sucrées au Canada. Les résultats variaient en fonction de l’indicateur de statut économique. Nous recommandons des interventions équitables pour réduire la consommation de ces boissons.

## Introduction

‘Sugary drinks’, defined as beverages with added sugars (‘sugar-sweetened beverages’ (SSB)) and beverages with natural intrinsic sugars (100% juice), contribute to excess dietary sugar intake (World Health Organization (WHO), 2015). Sugary drink consumption is associated with increased risk of obesity, type 2 diabetes, cardiovascular disease, cancer, disability-adjusted life years and mortality (Global Burden of Disease (GBD), 2018; Imamura et al., 2015; Makarem et al., 2018; Malik et al., 2013). Reducing consumption is a primary focus of global nutrition policies, including informational, financial, healthy default choices, and reduced availability interventions (Krieger et al., 2021). For example, the WHO recommends limiting free sugar to less than 10% of daily energy intake (WHO, 2015). Moreover, Canada’s updated 2019 Food Guide advises to ‘replace sugary drinks with water’, representing a change from the 2007 Canada’s Food Guide recommendations which included 100% juice as a fruit or vegetable serving (Health Canada, 2019b). In addition, Newfoundland and Labrador have included a 20-cent-per-litre sugary drink tax in the government’s proposed 2021–2022 budget, a first for Canada (Coady, 2021). There is a need to implement equitable interventions to reduce sugary drink consumption and associated health risks (Krieger et al., 2021).

In Canada, consumption of traditional sugary drinks (i.e., fruit drinks, regular soft drinks and 100% juice) has declined by one third between 2004 and 2015 (Czoli et al., 2019; Garriguet, 2019; Jones et al., 2019; Langlois et al., 2019). However, in 2015, sugary drinks contributed an average of 18% of total free sugar intake among Canadians (Liu et al., 2020) and were top dietary contributors of Canadians’ overall sugar intake across the lifespan (Kirkpatrick et al., 2019; Langlois et al., 2019). Of additional concern, purchases of novel types of SSB (e.g., energy and sport drinks) have increased among Canadians over time (Czoli et al., 2019), reflecting patterns observed in the United States (Drewnowski & Rehm, 2015; Han & Powell, 2013). Given the shifting consumption patterns, surveillance of sugary drinks overall and across beverage type is required to comprehensively assess population intake. Monitoring sugary drink consumption across socioeconomic position (SEP) is critical to understanding where inequities exist and their contribution to non-communicable disease risk. Low SEP groups have been found to consume more SSB and 100% juice in the USA (SEP: income and education) (Drewnowski & Rehm, 2015; Han & Powell, 2013), the United Kingdom (SEP: income) (Briggs et al., 2013) and Australia (SEP: income; SSB only) (Lal et al., 2017). In Canada, no differences in per capita SSB or 100% juice intake were observed across income (Jones et al., 2019), whereas intake across education and food security has yet to be established.

Comprehensive assessments of sugary drink consumption in Canada are needed. First, SEP indicators are not interchangeable proxies and descriptions of inequities associated with health knowledge (education), material resources (education, income and food security status) and material deprivation beyond accessing nutritious food (food security) are warranted (Braveman et al., 2005; Kirkpatrick et al., 2015). Moreover, age- and sex-specific patterns of sugary drink consumption across SEP should be considered. For example, compared to females, males have a higher prevalence of sugary drink consumption and consume more energy from these beverages in Canada (Garriguet, 2019; Jones et al., 2019; Langlois et al., 2019) and other high income countries (GBD, 2018). Finally, accounting for the episodic nature of beverage consumption is needed to better estimate social inequities in sugary drink consumption (Garriguet, 2019). Specifically, it is important to estimate the potential socioeconomic inequities in the prevalence of consumers, given that any consumption of these beverages is not recommended. Moreover, estimating socioeconomic inequities in mean energy intake from beverages allows us to assess the contribution of overall energy consumption among those who consumed (WHO, 2015).

Our objective was to estimate the sex-specific consumption of sugary drinks, SSB and 100% juice across SEP in a population-representative sample of Canadian children/adolescents and adults. This study fills a critical knowledge gap regarding the extent to which social inequities in sugary drink consumption exist in Canada.

## Methods

### Study population

We conducted a cross-sectional analysis of the nationally representative 2015 Canadian Community Health Survey–Nutrition (CCHS-N) Public Use Microdata File (Health Canada, 2017). The CCHS-N was conducted by Statistics Canada to assess dietary intakes of Canadians for the first time since 2004 using interviewer-administered 24-h dietary recalls using an adapted version of the Automated Multi-Pass Method from the United States Department of Agriculture (Statistics Canada, 2018). This survey used a multi-stage, cluster sampling approach to secure a sample of 20,487 Canadians aged 1 year and older living in private dwellings across the 10 provinces (61% response rate) (Health Canada, 2017).

We included single-day 24-h dietary recalls among Canadians 2 years and older (*n* = 20,115). We excluded respondents if they were breastfeeding or reported no energy intake (*n* = 200), or were missing information on income (*n* = 22), education (*n* = 41) or food security (*n* = 110). Our final analytic sample was 19,742 respondents. This study was approved by the Ethics Review Board at Public Health Ontario.

### Beverage types: sugary drinks, sugar-sweetened beverages (SSB) and 100% juice

We assessed sugary drink, SSB and 100% juice consumption separately. We applied Health Canada’s definition of sugary drinks (Health Canada, 2019a) to derive beverage types using Nutrition Survey System (NSS) codes linked to Canadian Nutrient File descriptions (see Appendix 1, Table 3). Sugary drink codes (*N* = 249) represent the sum of SSB (*N* = 190) and 100% juice (*N* = 59) codes. Our beverage categorization aligned with a previous Canadian study (Jones et al., 2019), with additional exclusion of beverages with sugar added by consumer (e.g., tea and coffee) and inclusion of sugary drinks consumed with alcohol. Diet/low-calorie beverages, infant formulas, functional beverages (e.g., meal replacements) and alcoholic beverages (e.g., pure alcohol, beer, wine, pre-mixed seltzers) were not included as sugary drinks.

Beverage consumption categories are not mutually exclusive because sugary drink consumers include consumers of either SSB or 100% juice or both SSB and 100% juice. We estimated sugary drink, SSB and 100% juice intake using two measures: (1) prevalence of consumers on a given day, defined as the proportion that reported consuming each beverage type on the day prior to the 24-h recall interview; and (2) mean energy intake (kilocalories (kcal)) among those who consumed each beverage type on a given day, estimated among those who consumed the day prior to the 24-h recall interview. To further quantify energy intake, we estimated the relative contribution of energy from each beverage type. We report averages; therefore, a single dietary recall was sufficient considering mean intake on a given day reflects mean usual intake (Garriguet, 2019).

### Socioeconomic position

The highest level of household education was categorized using four groups: ‘Less than high school’, ‘High school diploma’, ‘Certificate below bachelor’s degree’ (e.g., a trade, college or non-bachelor certificate) and ‘Bachelor degree or above’. Household food security status was assessed using eight questions for children/adolescents (aged 2–18) and ten for adults (aged 19 and over) with responses classified as either food secure (i.e., answered ‘yes’ to 0–1 questions about difficulty with income-related food access) or food insecure (moderate or severe, i.e., answered ‘yes’ to two or more questions about compromised quality/quantity or reduced food intake due to disrupted eating patterns) (Statistics Canada, 2018). Household income adequacy quintiles were derived based on the adjusted ratio of the respondents’ total household income reported in the previous 12 months to the low income cutoff corresponding to their household and community size (Statistics Canada, 2018).

### Covariates

We stratified our sample by sex (female or male) and age (children/adolescents, 2–18 years or adults, 19 years and older).

### Statistical analysis

We ran separate models for each outcome and SEP indicator. We applied modified Poisson regressions to estimate prevalence, prevalence differences and ratios (Appendix 1, Tables 6 and 7), and corresponding 95% confidence intervals (95% CI) across SEP using post-estimation marginal means analysis (Zou, 2004). Among those who consumed on a given day, we conducted linear regressions to estimate mean energy intake from each beverage type across SEP. For each beverage type, we estimated the relative (%) contribution of mean energy intake to the total energy intake from all sources. Primary analyses were unadjusted to describe consumption in a nationally representative sample (Conroy & Murray, 2020).

We conducted two sensitivity analyses. The residuals for the energy outcomes were right-skewed; therefore, we conducted square root-transformed regression and then back-transformed to original scale post-regression. Comparisons with the primary analysis revealed no differences in energy intake patterns (not shown); therefore, we reported untransformed energy outcomes to reflect reported energy intake from the CCHS-N (Lumley et al., 2002). Furthermore, we adjusted all models for dietary energy misreporting (under, over, plausible reporters, unclassified) to account for systematic error in self-reported dietary assessments (Appendix 2) (Garriguet, 2018).

We applied survey weights and bootstrap replicates (*N* = 500) provided by Statistics Canada in all models and used survey procedures ensuring results were representative of the Canadian population and appropriate variance estimation, respectively. Analyses were completed using SAS (v.9.4) and STATA (v.15).

## Results

### Prevalence of sugary drink, SSB and 100% juice consumers on a given day

**Fig. 1:**
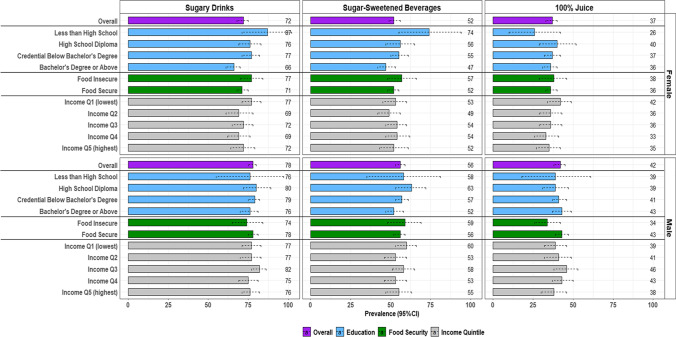
Sex-specific prevalence (%) of sugary drink consumption, on a given day, by beverage type and socioeconomic position in children/adolescents. Data source: 2015 Canadian Community Health Survey–Nutrition. Children/adolescents include respondents aged 2–18 years (females: 3050; males: 3064). SEP indicators include highest household educational attainment, household food security status and household income quintiles. Dotted lines represent 95% confidence intervals (CI). Prevalence values for the point estimates are included along the right-hand side of each figure. Figure created in R-Studio

Sex-specific prevalence of sugary drink, SSB and 100% juice consumers on a given day by SEP for children/adolescents is presented in Fig. 1 (estimates in Appendix 1, Table 4). Among *female and male children/adolescents*, the prevalence of consuming sugary drinks (females: 72% (95% CI: 68, 75); males: 78% (95% CI: 75, 80)), SSB (females: 52% (95% CI: 49, 56); males: 56% (95% CI: 53, 59)) and 100% juice (females: 37% (95% CI: 33, 40); males: 42% (95% CI: 38, 45)) on a given day was high. The absolute prevalence difference of sugary drink consumers ranged from 11 to 21 percentage-points higher among females in households with lower education (‘Less than high school’, ‘High school diploma’ and ‘Certificate below bachelor’s degree’) compared to ‘Bachelor’s degree or above’. Similarly, the prevalence of SSB consumers ranged from 8 to 27 percentage-points higher among females in households with lower education compared to those with ‘Bachelor’s degree or higher’. Among males, the prevalence of 100% juice consumers was 9 (95% CI: − 18, 0) percentage-points lower in food insecure compared to food secure households. In females, a lack of precision (i.e., uncertainty) was observed for the estimates related to higher prevalence of sugary drink and, separately, SSB consumers in food insecure compared to food secure and low compared to high income households (for sugary drinks only). In males, uncertainty surrounded higher prevalence of SSB consumers across education and comparing lowest to highest income. There was no clear pattern between the prevalence of 100% juice consumers and SEP in females and across education and income for males.

**Fig. 2:**
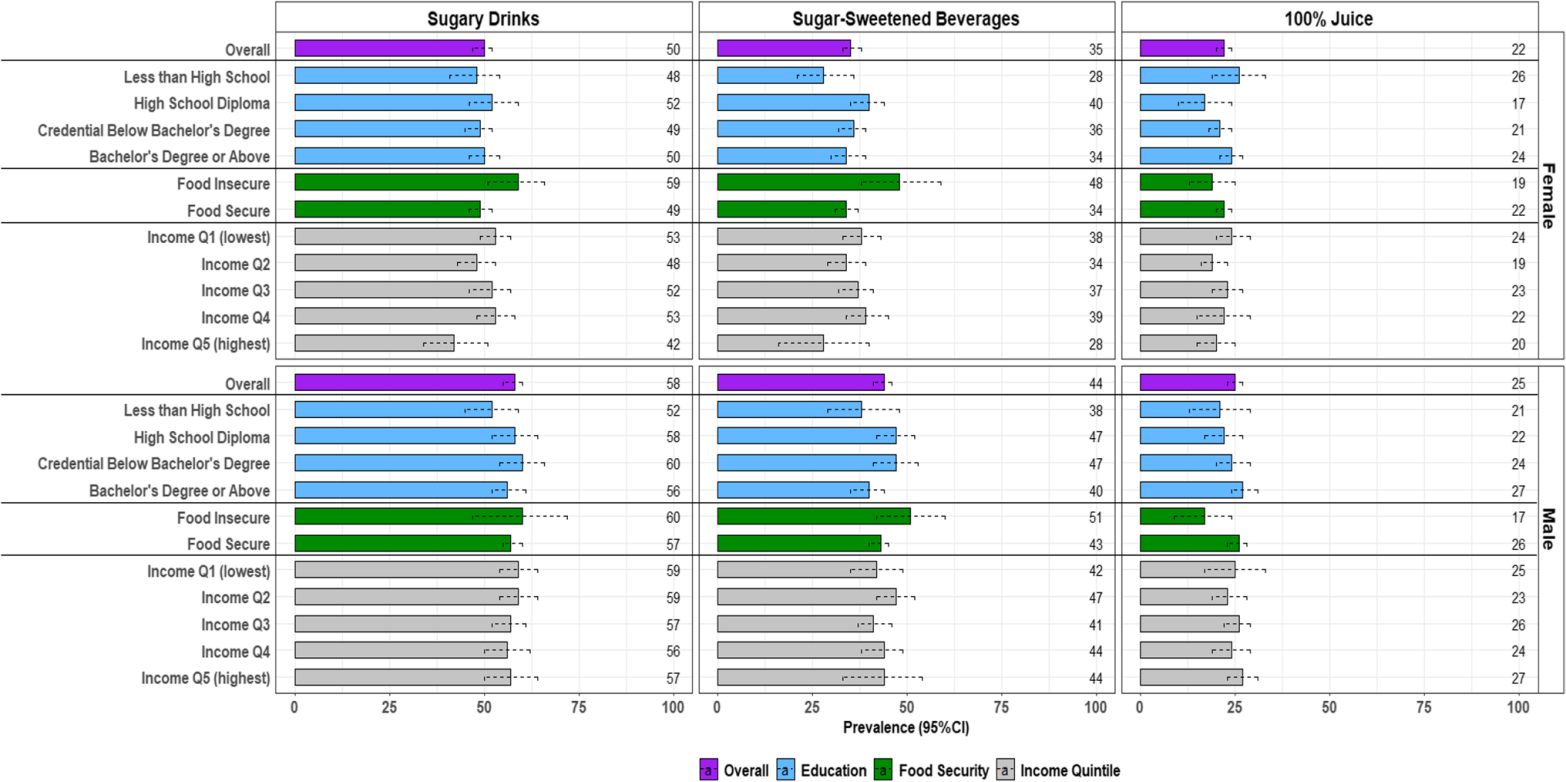
Sex-specific prevalence (%) of sugary drink consumption, on a given day, by beverage type and socioeconomic position in adults. Data source: 2015 Canadian Community Health Survey–Nutrition. Adults include respondents aged 19 years and older (females: 7219; males: 6409). SEP indicators include highest household educational attainment, household food security status and household income quintiles. Dotted lines represent 95% confidence intervals (CI). Prevalence values for the point estimates are included along the right hand side of each figure. Figure created in R-Studio

Sex-specific prevalence of sugary drink, SSB and 100% juice consumers on a given day by SEP in adults is presented in Fig. 2 (estimates in Appendix 1, Table 5). Among *female adults*, the prevalence of consuming on a given day was 50% (95% CI: 47, 52) for sugary drinks, 35% (95% CI: 33, 38) for SSB and 22% (95% CI: 20, 24) for 100% juice. On the absolute scale, the prevalence of sugary drink and, separately, SSB female adult consumers in food insecure households was 10 (95% CI: 1, 19) and 14 (95% CI: 2, 27) percentage-points higher than food secure households, respectively. Moreover, among female adults, consumption of sugary drinks among the lowest (Q1) and fourth income quintile (Q4), respectively, was 11 (95% CI: 1, 20) and 10 (95% CI: 2, 19) percentage-points higher compared to the highest (Q5) quintile. Uncertainty surrounded the higher prevalence estimates of increased sugary drink consumption comparing Q2 and Q3 income groups to Q5 and the higher estimates of SSB consumption among lower income groups. There was no clear pattern across education for SSB consumption and across all SEP indicators for 100% juice consumption in females.

Among *male adults*, the prevalence of consuming on a given day was 58% (95% CI: 55, 60) for sugary drinks, 44% (95% CI: 41, 46) for SSB and 25% (95% CI: 23, 27) for 100% juice. No differences were observed in the prevalence of sugary drink consumers across SEP. The prevalence of SSB consumers was between 7 and 8 percentage-points higher among males with ‘High school diploma’ and ‘Credential below bachelor’s degree’ compared to ‘Bachelor’s degree or above’ and 9 (95% CI: − 1, 19) percentage-points higher among males from food insecure compared to food secure households. Conversely, the prevalence of 100% juice consumers was 9 (95% CI: − 18, 0) percentage-points lower among male adults from food insecure compared to food secure households. There is additional evidence that low education males are consuming less 100% juice than their high SEP counterparts. Given the uncertainty around the estimates, additional confirmation is required to support interpretations.

Sex-specific prevalence ratios of sugary drink, SSB and 100% juice consumers on a given day by SEP for children/adolescents (Appendix 1, Table 6) and adults (Appendix 1, Table 7) followed the same patterns described for the absolute differences.

### Mean energy intake from sugary drinks, SSB and 100% juice, among consumers on a given day

Sex-specific mean energy intake from beverages among children/adolescents who consumed sugary drinks, SSB and 100% juice on a given day by SEP is presented in Table 1. Among *female children/adolescents*, mean daily energy intake was 211 kcal (95% CI: 200, 223) from sugary drinks, 197 kcal (184, 210) from SSB and 132 kcal (95% CI: 122, 143) from 100% juice, representing 12%, 11% and 8% of overall energy intake among consumers on a given day, respectively. No social inequities in mean energy intake were observed.

**Table 1:** Sex-specific mean energy intake (kcal) and mean differences from sugary drinks by socioeconomic position among children/adolescent consumers on a given day

	Sugary drinks				Sugar-sweetened beverages				100% Juice			
	*N*	Mean	Mean difference	% Daily energy	*N*	Mean	Mean difference	% Daily energy	*N*	Mean	Mean difference	% Daily energy
		(95% CI)	(95% CI)			(95% CI)	(95% CI)			(95% CI)	(95% CI)	
**Females (2–18 years)**												
Overall population	2200	211 (200, 223)		12%	1664	197 (184, 210)		11%	1051	132 (122, 143)		8%
Highest household education												
Less than high school	76	201 (95, 306)	− 6 (− 113, 100)	12%	59	180 (100, 260)	− 13 (− 97, 71)	11%	29	155 (71, 239)	31 (− 53, 114)	8%
High school diploma	359	233 (201, 266)	26 (− 11, 63)	13%	288	211 (182, 241)	18 (− 18, 54)	12%	153	147 (98, 196)	22 (− 26, 70)	8%
Credential below bachelor’s degree	899	210 (193, 226)	3 (− 20, 25)	12%	684	199 (179, 220)	6 (− 22, 34)	11%	413	135 (123, 147)	10 (− 8, 29)	7%
Bachelor’s degree or above	866	207 (191, 223)	Ref	12%	633	193 (175, 211)	Ref	11%	456	125 (111, 138)	Ref	8%
Household food security status												
Food insecure	281	220 (192, 248)	9 (− 21, 39)	13%	223	208 (173, 243)	12 (− 24, 48)	11%	119	136 (117, 155)	4 (− 21, 28)	8%
Food secure	1919	210 (198, 223)	Ref	12%	1441	196 (183, 210)	Ref	11%	932	132 (120, 144)	Ref	8%
Household income quintile												
Q1, lowest	436	193 (174, 211)	− 9 (− 39, 20)	11%	325	175 (153, 197)	− 19 (− 51, 13)	10%	205	135 (116, 155)	9 (− 21, 39)	8%
Q2	425	233 (200, 265)	31 (− 15, 76)	13%	314	220 (183, 256)	26 (− 19, 71)	12%	214	150 (120, 180)	24 (− 13, 60)	8%
Q3	502	221 (202, 241)	19 (− 14, 52)	13%	380	211 (189, 232)	17 (− 15, 50)	12%	241	125 (110, 140)	− 1 (− 31, 29)	7%
Q4	440	207 (175, 239)	5 (− 29, 40)	12%	341	188 (151, 225)	− 5 (− 48, 37)	11%	212	121 (102, 140)	− 6 (− 32, 21)	7%
Q5, highest	397	202 (177, 227)	Ref	11%	304	193 (169, 218)	Ref	10%	179	126 (102, 151)	Ref	7%
**Males (2–18 years)**												
Overall population	2359	252 (234, 270)		12%	1800	238 (215, 260)		11%	1190	149 (136, 163)		7%
Highest household education												
Less than high school	78	317 (232, 402)	65 (− 26, 157)	15%	63	301 (190, 411)	59 (− 61, 180)	14%	37	171 (84, 257)	21 (− 71,113)	7%
High school diploma	379	268 (228, 308)	16 (− 35, 67)	14%	313	239 (200, 278)	− 2 (− 52, 48)	12%	166	166 (116, 216)	16 (− 40, 73)	9%
Credential below bachelor’s degree	988	242 (217, 268)	− 10 (− 50, 30)	12%	755	230 (204, 257)	− 11 (− 58, 36)	11%	484	143 (124, 162)	− 7 (− 36, 23)	7%
Bachelor’s degree or above	914	252 (223, 282)	Ref	12%	669	241 (201, 282)	Ref	11%	503	149 (133, 166)	Ref	7%
Household food security status												
Food insecure	316	279 (236, 323)	31 (− 23, 85)	13%	249	263 (204, 322)	29 (− 46, 104)	12%	134	160 (126, 195)	12 (− 21, 45)	7%
Food secure	2043	248 (227, 270)	Ref	12%	1551	234 (206, 262)	Ref	11%	1056	148 (135, 161)	Ref	7%
Household income quintile												
Q1, lowest	459	257 (229, 285)	− 9 (− 75, 56)	13%	363	223 (191, 254)	− 42 (− 104,21)	11%	225	165 (142, 187)	13 (− 34, 59)	8%
Q2	446	228 (202, 253)	− 39 (− 103, 26)	12%	347	214 (183, 244)	− 51 (− 115,14)	10%	203	150 (127, 174)	− 2 (− 48, 45)	8%
Q3	570	272 (226, 317)	6 (− 82, 93)	13%	421	269 (208, 330)	5 (− 85, 94)	13%	292	145 (131, 158)	− 7 (− 51, 36)	7%
Q4	483	231 (199, 263)	− 35 (− 92, 22)	11%	356	218 (188, 248)	− 47 (− 110,17)	10%	265	136 (106, 166)	− 16 (− 60,28)	6%
Q5, highest	401	266 (201, 331)	Ref	12%	313	264 (202, 327)	Ref	11%	205	152 (109, 195)	Ref	7%

Data source: 2015 Canadian Community Health Survey–Nutrition. Children/adolescents include respondents aged 2–18 years (females: 3050; males: 3064). Mean energy is reported as ‘energy on a given day (kcal)’. SEP indicators include highest household educational attainment, household food security status and household income quintiles. Mean difference (MD) represents the absolute difference in mean intake and associated 95% CI across SEP (education, food security, income quintile). ‘% daily energy’ represents the mean contribution of daily energy from beverages among consumers on the previous day to the 24-h recall. '*N*' represents the number of respondents who reported consuming each beverage type across sex/age/and SEP indicators

Among *male children/adolescents*, mean energy intake was 252 kcal (95% CI: 234, 270) from sugary drinks, 238 kcal (95% CI: 215, 260) from SSB and 149 kcal (95% CI: 136, 163) from 100% juice, representing 12%, 11% and 7% of overall energy intake among consumers, respectively. While the relative contribution of overall energy intake from sugary drinks and SSB was 3% higher among male children/adolescents with ‘Less than high school’ compared to ‘Bachelor’s degree or above’, uncertainty surrounded the mean differences. There were no clear patterns observed across SEP for 100% juice consumers.

Sex-specific mean energy intake among adult consumers of sugary drinks, SSB and 100% juice on a given day by SEP is presented in Table 2. Among *female adults*, mean energy intake was 186 kcal (95% CI: 175, 198) from sugary drinks, 193 kcal (95% CI: 180, 206) from SSB and 113 kcal (95% CI: 102, 124) from 100% juice, representing 11%, 11% and 6% of overall energy intake among consumers, respectively. Food insecure consumers, on average, consumed 27 kcal (95% CI: 3, 51) more energy from sugary drinks. Similar inequities were observed for SSB consumption across food security; however, additional verification is required due to uncertainty related to small sample size. One-hundred percent juice consumers with ‘Less than high school’ consumed, on average, 35 kcal (95% CI: 2, 67) more energy from 100% juice than female consumers with ‘Bachelor’s degree or above’.

**Table 2:** Sex-specific mean energy intake (kcal) and mean differences from sugary drinks by socioeconomic position among adult consumers on a given day

	Sugary drinks				Sugar-sweetened beverages				100% Juice			
	*N*	Mean	Mean difference	% Daily energy	*N*	Mean	Mean difference	% Daily energy	*N*	Mean	Mean difference	% Daily energy
		(95% CI)	(95% CI)			(95% CI)	(95% CI)			(95% CI)	(95% CI)	
**Females (≥ 19 years)**												
Overall population	3661	186 (175, 198)		11%	2630	193 (180, 206)		11%	1556	113 (102, 124)		6%
Highest household education												
Less than high school	443	197 (157, 237)	15 (− 21, 50)	12%	290	207 (154, 260)	14 (− 29, 56)	13%	204	137 (106, 167)	35 (2, 67)	9%
High school diploma	739	168 (151, 184)	− 15 (− 40, 11)	10%	551	174 (154, 194)	− 19 (− 54, 16)	11%	290	109 (93, 125)	7 (− 12, 25)	6%
Credential below bachelor’s degree	1323	199 (181, 217)	17 (− 3, 36)	11%	971	200 (183, 218)	7 (− 16, 31)	11%	544	122 (100, 144)	20 (− 5, 45)	6%
Bachelor’s degree or above	1156	182 (167, 198)	Ref	10%	818	193 (171, 216)	Ref	11%	518	102 (93, 111)	Ref	6%
Household food security status												
Food insecure	408	211 (186, 236)	27 (3, 51)	13%	324	214 (187, 240)	24 (− 2, 49)	12%	136	111 (71, 150)	− 2 (− 50, 45)	6%
Food secure	3253	184 (172, 196)	Ref	11%	2306	190 (177, 203)	Ref	11%	1420	113 (99, 127)	Ref	6%
Household income quintile												
Q1, lowest	885	194 (176, 213)	8 (− 21, 36)	12%	641	200 (178, 221)	− 4 (− 36, 28)	12%	378	111 (96, 126)	2 (− 22, 25)	6%
Q2	833	169 (149, 190)	− 17 (− 48, 13)	10%	588	166 (146, 186)	− 37 (− 78, 4)	10%	358	125 (102, 147)	15 (− 26, 57)	7%
Q3	784	178 (152, 204)	− 8 (− 42, 25)	10%	563	185 (155, 214)	− 19 (− 52, 14)	11%	325	106 (92, 121)	− 3 (− 25, 19)	6%
Q4	616	203 (175, 232)	17 (− 18, 51)	11%	445	211 (185, 237)	7 (− 32, 47)	12%	273	114 (80, 147)	4 (− 20, 28)	6%
Q5, highest	543	186 (165, 208)	Ref	11%	393	204 (173, 234)	Ref	12%	222	109 (85, 134)	Ref	6%
**Males (≥ 19 years)**												
Overall population	3545	243 (230, 257)		10%	2648	246 (230, 261)		10%	1487	134 (123, 145)		6%
Highest household education												
Less than high school	341	238 (155, 320)	22 (− 61, 106)	11%	240	257 (171, 344)	41 (− 48, 130)	12%	149	120 (45, 196)	− 11 (− 96, 73)	6%
High school diploma	721	283 (248, 319)	68 (29, 107)	12%	568	283 (244, 322)	67 (21, 113)	12%	259	147 (122, 173)	16 (− 16, 48)	6%
Credential below bachelor’s degree	1322	255 (230, 280)	39 (10, 69)	11%	1040	253 (224, 282)	36 (5, 68)	10%	500	134 (110, 158)	3 (− 32, 37)	5%
Bachelor’s degree or above	1161	216 (199, 232)	Ref	10%	800	217 (195, 238)	Ref	9%	579	131 (114, 148)	Ref	6%
Household food security status												
Food insecure	314	327 (217, 437)	91 (− 30, 212)	14%	260	336 (215, 457)	99 (− 34, 232)	14%	92	141 (104, 178)	8 (− 30, 45)	6%
Food secure	3231	236 (219, 254)	Ref	10%	2388	237 (217, 257)	Ref	10%	1395	134 (122, 145)	Ref	6%
Household income quintile												
Q1, lowest	619	262 (215, 310)	20 (− 41, 80)	12%	471	277 (205, 350)	37 (− 59, 133)	12%	241	160 (126, 194)	33 (− 12, 77)	8%
Q2	710	249 (207, 291)	6 (− 41, 53)	11%	547	244 (191, 298)	4 (− 49, 57)	11%	287	134 (119, 149)	7 (− 20, 34)	6%
Q3	793	233 (207, 259)	− 10 (− 44, 24)	10%	578	241 (214, 269)	1 (− 42, 45)	10%	346	127 (99, 156)	0 (− 27, 27)	6%
Q4	671	232 (208, 256)	− 11 (− 44, 22)	10%	511	231 (202, 260)	− 10 (− 48, 29)	10%	276	126 (101, 152)	− 1 (− 28, 26)	5%
Q5, highest	752	243 (216, 270)	Ref	10%	541	240 (203, 277)	Ref	9%	337	127 (107, 148)	Ref	5%

Data source: 2015 Canadian Community Health Survey–Nutrition. Adults include respondents aged 19 years and older (females: 7219; males: 6409). Mean energy is reported as ‘energy on a given day (kcal)’. SEP indicators include highest household educational attainment, household food security status and household income quintiles. Mean difference (MD) represents the absolute difference in mean intake and associated 95% CI across SEP (education, food security, income quintile). ‘% daily energy’ represents the mean contribution of daily energy from beverages among consumers on the previous day to the 24-h recall. '*N*' represents the number of respondents who reported consuming each beverage type across sex/age/and SEP indicators.

Among *male adults*, mean energy intake from beverages was 243 kcal (95% CI: 230, 257) from sugary drinks, 246 kcal (95% CI: 230, 261) from SSB and 134 kcal (95% CI: 123, 145) from 100% juice, representing 10%, 10% and 6% of overall daily energy intake, respectively. Sugary drink consumers with ‘High school diploma’ and ‘Credential below bachelor’s degree’ consumed, on average, 68 and 39 kcal more energy from sugary drinks compared to those with ‘Bachelor’s degree or above’. Similar patterns were observed for SSB energy intake across education. While the relative contribution of energy intake among sugary drink and, separately, SSB consumers was 4% higher among food insecure compared to food secure male adults, the uncertainty surrounding the mean differences requires further verification. No patterns in energy intake were observed among male adult 100% juice consumers across SEP.

### Accounting for systematic error related to energy misreporting

Adjusted intake patterns for dietary misreporting were consistent with the primary analyses (available upon request).

## Discussion

This study identified sex-specific social inequities in sugary drink and SSB consumption in a nationally representative sample of Canadians, but patterns were inconsistent for 100% juice. Low education was associated with higher prevalence of sugary drink and SSB consumption on a given day among female children/adolescents, while food insecurity and low income were associated with a higher prevalence of sugary drink consumption in female adults. Among male children/adolescents and adults, a lower prevalence of 100% juice consumption on a given day was observed among food insecure compared to food secure groups. Among consumers on a given day, higher mean energy intake from sugary drinks and SSB was observed across food security in female adults and education in male adults. Among female adults who consumed 100% juice, low education was associated with higher energy intake. Across all SEP groups, Canadians reported a high prevalence of sugary drink, SSB and 100% juice consumption on a given day and have mean energy intake levels associated with poor health outcomes (WHO, 2015). Together our findings indicate the need for pro-equity interventions to reduce consumption among Canadians.

Population-level interventions such as informational and financial policies have potential to reduce unhealthy beverage consumption and may influence manufacturers to produce healthier products (Krieger et al., 2021). Informational interventions, including Canada’s ‘replace sugary drinks with water’ messaging (Health Canada, 2019b) or traffic-light labelling interventions (von Philipsborn et al., 2019), are important, but likely insufficient for achieving this goal. Moreover, informational interventions may impose literacy burdens on consumers, limiting access for certain groups (Krieger et al., 2021). Taxation policies are considered pro-equity interventions given the beneficial impact across subpopulations. Internationally, SSB taxation policies are associated with reduced SSB sales concentrated among lower SEP groups (Krieger et al., 2021; Popkin & Ng, 2021). Modelling studies have demonstrated the potential for beverage taxation policies to reduce social inequities in non-communicable diseases and increase healthcare cost savings concentrated among lower SEP groups (Kao et al., 2020; Lal et al., 2017). Finally, limiting SSB availability in subsidized food programs may improve diet quality of recipients, particularly when paired with incentives to increase fruit/vegetable purchases (Krieger et al., 2021).

Establishing baseline levels of beverage consumption by age, sex and SEP is essential for guiding the choice and design of interventions to equitably reduce sugary drink consumption and associated non-communicable disease in Canada (Popkin & Ng, 2021). Few studies have examined the association between SEP and sugary drink consumption, an important risk factor for health outcomes (Briggs et al., 2013; Han & Powell, 2013; Jones et al., 2019; Lal et al., 2017). A previous Canadian study using the CCHS-N reported no differences in per capita energy intake from SSB across income levels (Jones et al., 2019). Our study expanded upon this analysis by assessing total sugary drinks, incorporating the episodic nature of beverage consumption, disaggregating findings by sex, describing outcomes across income quintiles and examining additional household SEP measures (educational attainment and food security status). In our study, more patterns emerged for food security status and educational attainment compared to income for sugary drinks and SSB consumption, highlighting the importance of describing patterns across multiple SEP indicators in heath research (Braveman et al., 2005). To our knowledge, this is the first Canadian study to include food security status as an independent exposure of sugary drink intake incorporating work that identified food security status as a strong predictor of differential nutrient intake in Canada (Kirkpatrick et al., 2015). Our findings related to SSB consumption across education are consistent with associations found in the USA (Han & Powell, 2013). More income-related inequities in SSB consumption were observed in the USA, the UK and Australia compared to our study (Briggs et al., 2013; Han & Powell, 2013; Lal et al., 2017), which may be related to different subgroups and income measures analyzed across studies.

Our study found no clear pattern between SEP and 100% juice consumption consistent with a previous Canadian assessment of 100% juice energy intake across income quartiles (Jones et al., 2019). In our study, energy intake among female adult consumers from 100% juice was higher in the lowest education group. Conversely, males had a lower prevalence of 100% juice consumption in food insecure compared to secure groups. Despite the variability in our findings across 100% juice, monitoring social inequities in 100% juice consumption is important, as these have been observed in the USA (Drewnowski & Rehm, 2015) and the UK (Briggs et al., 2013). Differential uptake of Canada’s Food Guide recommendations to replace sugary drinks with water across SEP (Health Canada, 2019b), coupled with the lower cost of 100% juice compared to whole fruits and vegetables, could generate unintended inequities in 100% juice consumption (Darmon & Drewnowski, 2015).

This study has limitations. Our study relies on data collected in 2015 and may not reflect temporal changes to dietary intake patterns; however, we leveraged the most recent population-level nutrition data available. Social desirability bias in self-reported responses may have led to underestimates of consumption in some groups (e.g., females and higher SEP), which would bias observed social inequities towards the null (Garriguet, 2018). For example, self-reported beverage intakes in the CCHS-N were consistently lower than Canadian beverage sales over the same time period (Czoli et al., 2019). Therefore, we adjusted all models for energy misreporting status in our sensitivity analyses, which had minimal impact on our findings. Low sample sizes across SEP groups likely resulted in insufficient power to detect significant associations, particularly in children/adolescents; however, sole reliance on statistical significance for interpretations in epidemiology is incomplete (Wasserstein & Lazar, 2016). Analyses of single-day dietary recall estimates are prone to random error associated with within-person variability, which can be accounted for through analyses of multiple dietary recalls, such as the National Cancer Institute’s method for estimating usual dietary intake (Tooze et al., 2006). This bias may have contributed to the reduced precision of mean energy intake estimates across SEP due to smaller sample sizes in these analyses; however, for means a single 24-h recall is sufficient and our approach allowed us to assess our specific a priori research questions related to socioeconomic differences in the prevalence, per-consumer energy intake and relative contribution of total energy, on a given day. Twenty-four-hour recalls are prone to systematic error in energy reporting, which have been accounted for in the study design and sensitivity analysis (Health Canada, 2017). Reliance on the CNF descriptions to categorize beverages may have led to misclassification of certain codes; however, our categorizations are generally consistent with a previous study using the CCHS-N (Jones et al., 2019). Finally, inclusion of only pre-sweetened beverages likely underestimated consumption, though population-level interventions generally target pre-made items (Popkin & Ng, 2021; von Philipsborn et al., 2019).

This study’s strengths include analyzing three distinct primary exposures of household SEP to describe SEP-specific consumption patterns (Braveman et al., 2005; Kirkpatrick et al., 2015). We observed differential consumption patterns by sex, highlighting the importance of assessing sex-specific associations in health research (Johnson et al., 2009). We applied methods to analyze episodically consumed beverages (Han & Powell, 2013) and analyzed separate consumption patterns for sugary drinks, SSB and 100% juice associated with free, added and natural sugars, respectively (WHO, 2015). Finally, we included a comprehensive assessment of traditional and novel beverage types (Czoli et al., 2019; Jones et al., 2019).

## Conclusion

To our knowledge, this is the first study to describe age- and sex-specific sugary drink, SSB and 100% juice consumption patterns across multiple indicators of SEP in a representative sample of Canadians. Future research on the effectiveness of interventions to equitably reduce sugary drink consumption in the Canadian context is warranted.

## Contributions to knowledge

What does this study add to existing knowledge?

- This study adds to a growing body of evidence highlighting population and socioeconomic patterns of beverage consumption by including multiple socioeconomic indicators (education, food security status and income adequacy quintile) to describe differential patterns of beverage intake. In addition, population-level intake on a given day was high across all SEP groups by age and sex.
  What are the key implications for public health interventions, practice or policy?
- The design and implementation of pro-equity population-level interventions that reduce sugary drink consumption in the Canadian population are warranted.
- Informational, financial, healthy defaults and limited availability interventions should be considered in reference to baseline consumption patterns for the design and implementation of sustainable strategies targeting food and social environments in Canada.

## Data Availability

The data that support the findings of this study are available from Statistics Canada upon request (https://www150.statcan.gc.ca/n1/en/catalogue/82M0024X).

## Appendix 1

**Table 3:** Nutrient Survey System codes and Canadian Nutrient File descriptions for beverage groupings categorized as sugary drinks, sugar-sweetened beverages and 100% juice

Nutrient Survey System code	Canadian Nutrient File description
100% Juice (beverage types: sugary drink, 100% juice)	
1485	Acerola juice, raw
1495	Apple juice, canned or bottled, without added vitamin C
1497	Apple juice, frozen concentrate, diluted, without added vitamin C
1572	Grapefruit juice, white, raw
1576	Grape juice, canned or bottled, without added vitamin C
1589	Lemon juice, raw
1590	Lemon juice, canned or bottled
1591	Lemon juice, frozen
1594	Lime juice, raw
1595	Lime juice, canned or bottled
1619	Orange juice, raw
1620	Orange juice, chilled, includes from concentrate
1622	Orange grapefruit juice, canned
1624	Tangerine (mandarin) juice, raw
1631	Passion fruit juice, purple, raw
1632	Passion fruit juice, yellow, raw
1657	Pineapple juice, canned, added vitamin C
1659	Pineapple juice, frozen concentrate, diluted
1673	Prune juice, canned
1716	Grapefruit juice, canned, no added sugar
1723	Orange juice, canned
1725	Orange juice, frozen concentrate, diluted
1752	Apple juice, canned or bottled, added vitamin C
1754	Apple juice, frozen concentrate, diluted, added vitamin C
2312	Carrot juice, canned
2464	Juice, tomato, canned
2473	Vegetable juice cocktail, canned
2868	Juice, tomato clam cocktail, canned
5287	Juice drink, mixed vegetable and fruit
5389	Blackberry juice, canned
5472	Orange-strawberry-banana juice
5586	Juice, tomato and vegetable, low sodium
5593	Cranberry juice, unsweetened
6203	Orange juice, chilled, includes from concentrate, fortified with added calcium and vitamin D
6287	Juice, tomato, canned, no salt added
6440	Grapefruit juice, pink, raw
6660	Grape juice, canned or bottled, unsweetened, with added vitamin C
6661	Pomegranate juice, ready-to-drink
7051	Orange pineapple juice
7224	Beverage, coconut water, unsweetened, ready-to-drink
7419	Apple juice, canned or bottled, unsweetened, calcium and vitamins C and D added
7421	Vegetable juice cocktail, canned, low sodium
7573	Orange juice, frozen concentrate, diluted, with added calcium and vitamin D
404283	Fruit juice blend, 100% juice, with vitamins and minerals
501855	Orange and banana juice
501857	Pineapple-grapefruit juice, fresh
501862	Pineapple-orange juice, NFS, includes from concentrate
501866	Pineapple-orange juice, frozen, diluted with water
501927	Fruit juice, NFS (mixed fruit juices)
502123	Mixed vegetable juice (vegetables other than tomato)
504002	Apple cider
504189	Pineapple juice-non-citrus juice blend, unsweetened, with added vitamin C
504190	Pineapple, orange and banana juice
504388	Fruit juice blend, 100% juice, with added vitamin C
504477	Orange juice, frozen concentrate, unsweetened, diluted
504478	Orange juice, frozen concentrate, with calcium and vit. D added, diluted
504731	Orange juice, frozen concentrate, diluted with 4 parts water
504732	Orange juice, frozen concentrate, diluted with 3.5 parts water
505135	Orange juice, NFS
Sugar-Sweetened Fruit Drink (beverage type: sugary drink, sugar-sweetened beverage)	
1570	Grapefruit juice, canned, sweetened
1625	Tangerine (mandarin) juice, canned, sugar added
1629	Papaya nectar, canned
1644	Peach nectar, canned
1652	Pear nectar, canned
1694	Apricot nectar, canned
1717	Grapefruit juice, frozen concentrate, diluted
1720	Grape juice, frozen concentrate, sugar and vitamin C added, diluted
2885	Juice drink, cranberry and apricot, bottled
2889	Juice drink, citrus fruit, frozen concentrate, water added
2893	Lemonade, white, frozen concentrate, water added
2895	Limeade, frozen concentrate, water added
2904	Juice drink, orange and apricot, canned
2922	Juice drink, fruit punch, frozen concentrate, water added
2940	Lemonade, pink, frozen concentrate, water added
2954	Juice drink, cranberry-apple, vitamin C added, bottled
2955	Juice drink, cranberry-grape, vitamin C added, bottled
2956	Juice, cocktail, cranberry, vitamin C added, bottled
2958	Drink, fruit punch flavour, vitamin C added, powder, water added
2959	Drink, fruit punch, vitamin C added, ready-to-drink
2960	Juice drink, grape, vitamin C added, canned
2961	Drink, grape, vitamin C added, canned
2965	Drink, lemonade flavour, vitamin C added, powder, water added
2967	Drink, orange, vitamin C added, canned
2968	Juice drink, pineapple and grapefruit, vitamin C added, canned
2969	Juice drink, pineapple and orange, vitamin C added, canned
2972	Drink, orange flavour, vitamin C added, powder, water added
2974	Drink, orange, vitamin C added, frozen concentrate, water added
2976	Juice, cocktail, cranberry, vitamin C added, frozen concentrate, water added
2981	Drink, fruit punch flavour, powder, water added
2983	Drink, lemonade flavour, powder, water added
5424	Drink, breakfast type, orange, ready-to-drink
5628	Cocktail mix, non-alcoholic, concentrated, frozen
6204	Guava, nectar, canned
6205	Mango, nectar, canned
6437	Drink, fruit punch, frozen concentrate, water added
6470	Juice drink, orange
6662	Juice, apple and grape, with added vitamin C
7055	Drink, fruit flavour, vitamin C added, ready-to-drink
7070	Juice drink, fruit, ready-to-drink
7230	Juice drink, orange, calorie-reduced
404292	Juice drink, fruit, without added vitamin C, ready-to-drink
501853	Grapefruit and orange juice, fresh, with sugar
501935	Prune juice, with sugar
501936	Banana nectar
501937	Cantaloupe nectar
501940	Passion fruit nectar
501941	Soursop (Guanabana) nectar
502464	Fruit punch, made with fruit juice and soda
502465	Fruit punch, made with soda, fruit juice, and sherbet or ice cream
502468	Lemonade, frozen, diluted with water
502469	Lemon-limeade
502470	Limeade, frozen concentrate, diluted
502472	Citrus fruit juice drink, frozen concentrate, diluted (40–50% fruit juice)
502479	Fruit-flavoured drink, made from powdered mix (lemonade)
502480	Lemonade, drink, powder, with sugar and vitamin C added, water added
504387	Daiquiri mix, frozen concentrate, diluted
504510	Pina Colada, non-alcoholic
504558	Shirley Temple
504686	Carbonated citrus juice drink
504733	Juice drink, fruit punch, frozen concentrate, diluted with 3.5 parts water
504734	Juice drink, fruit punch, frozen concentrate, diluted with 4 parts water
504735	Juice drink, fruit punch, frozen concentrate, diluted with 5 parts water
505134	Grapefruit juice, frozen (reconstituted with water)
505188	Carbonated juice drink, NS as to type of juice
505189	Carbonated noncitrus juice drink
Regular soft drink (beverage type: sugary drink, sugar-sweetened beverage)	
2854	Carbonated drinks, cream soda
2855	Carbonated drinks, ginger ale
2856	Carbonated drinks, grape soda
2857	Carbonated drinks, lemon-lime soda
2858	Carbonated drinks, orange soda
2859	Carbonated drinks, pepper type
2860	Carbonated drinks, tonic water (quinine)
2861	Carbonated drinks, root beer
2920	Carbonated drinks, cola, fast food cola
4980	Carbonated drinks, cola, decaffeinated
5288	Carbonated drinks, cola
5293	Carbonated drinks, chocolate
7429	Carbonated drinks, lemon-lime soda, with caffeine
Sugar-sweetened milk (beverage type: sugary drink, sugar-sweetened beverage)	
55	Eggnog, 7% M.F., Canadian product, 4% to 8% M.F.
69	Milk, fluid, chocolate, whole
70	Milk, fluid, chocolate, partly skimmed, 2% M.F.
75	Milk shake, chocolate, thick
76	Milk shake, vanilla, thick
2863	Beverage mix, carob flavour, powder, with whole milk
2865	Beverage mix, chocolate flavour, powder, with whole milk
2896	Malted milk, natural flavour, enriched powder
2899	Malted milk, natural flavour, powder, with whole milk
2900	Malted milk, chocolate flavour, enriched powder
2903	Malted milk, chocolate flavour, powder, with whole milk
2905	Milk shake (fast food), chocolate
2906	Milk shake (fast food), vanilla
2908	Strawberry flavour mix, powder, with whole milk
2932	Milk shake (fast food), strawberry
4711	Milk, fluid, chocolate, partly skimmed, 1% M.F.
5268	Beverage mix, carob flavour, powder, with 2% M.F. milk
5269	Beverage mix, chocolate flavour, powder, with 2% M.F. milk
5273	Malted milk, natural flavour, powder, with 2% M.F. milk
5275	Malted milk, chocolate flavour, powder, with 2% M.F. milk
5276	Strawberry flavour mix, powder, with 2% M.F. milk
5589	Chocolate flavour drink, whey and milk-based
6636	Hot chocolate, mix, powder, prepared with 2% milk
500024	Chocolate flavour mix beverage, powder, milk added, NS as to type of milk
500025	Chocolate flavour mix beverage, whole milk added
500026	Chocolate, flavour mix beverage, powder, 1% milk added
500027	Chocolate flavour mix beverage, skim milk added
500035	Malted milk, natural flavour, enriched, powder, 2% milk added
500036	Malted milk, chocolate, enriched, powder, milk added
500037	Malted milk, NS as to flavour, enriched, powder, milk added
500038	Eggnog, made with 2% milk
500039	Milk shake, NS as to flavour or type
500040	Milk shake, homemade or fountain-type, NS as to flavour
500041	Milk shake with malt (malted milk with ice cream)
500042	Milk shake, restaurant type, NS as to flavour (thick shake mix, milk added)
500043	Milk-based fruit drink
500064	Ice cream soda, flavours other than chocolate (root beer float)
500065	Ice cream soda, chocolate (root beer float)
502759	Chocolate flavour mix beverage, powder, 2% milk added
502760	Chocolate syrup, 2% milk added
502770	Milk, flavours other than chocolate, 2% milk-based (strawberry, vanilla, powder and syrup)
502783	Milk shake, restaurant type, chocolate, thick
502784	Milk shake, restaurant type, vanilla, thick
504172	Milk shake, homemade or fountain-type, chocolate
504173	Milk shake, homemade or fountain-type, flavours other than chocolate
504359	Chocolate syrup, skim milk added
504695	Chocolate syrup, 1% milk added
504696	Chocolate syrup, whole milk added
504974	Milk, chocolate, NFS
504976	Chocolate syrup, milk added, NS as to type of milk
504977	Milk, flavours other than chocolate, NFS (strawberry, vanilla, powder and syrup)
504978	Milk, flavours other than chocolate, whole milk-based (strawberry, vanilla, powder and syrup)
504979	Milk, flavours other than chocolate, 1% milk-based (strawberry, vanilla, powder and syrup)
504980	Milk, flavours other than chocolate, skim milk-based (strawberry, vanilla, powder and syrup)
Sugar-sweetened plant-based beverage (beverage type: sugary drink, sugar-sweetened beverage)	
4780	Plant-based beverage, rice, enriched
5241	Plant-based beverage, soy, original and vanilla, unenriched
5429	Plant-based beverage, soy, unenriched, chocolate
6329	Plant-based beverage, soy, enriched, chocolate
6331	Plant-based beverage, soy, enriched, all flavours, low fat
6332	Plant-based beverage, soy, enriched, all flavours, fat free
6666	Plant-based beverage, soy, enriched, with omega-3 fatty acids added
6720	Plant-based beverage, soy, enriched, all flavours
6784	Plant-based beverage, soy, enriched, all flavours, reduced fat
7225	Plant-based beverage, almond, enriched, sweetened, vanilla flavoured
7226	Plant-based beverage, almond, enriched, sweetened, chocolate flavoured
7478	Plant-based beverage, coconut, enriched, sweetened, all flavours
7480	Plant-based beverage, cashew, enriched, sweetened
7570	Plant-based beverage, coconut, unenriched, sweetened, all flavours
Sugar-sweetened smoothie (beverage type: sugary drink, sugar-sweetened beverage)	
504145	Fruit smoothie drink, made with fruit or fruit juice only (no dairy products)
504171	Milk fruit drink (smoothie)
504981	Fruit smoothie drink, NFS
Sugar-sweetened coffee (beverage type: sugary drink, sugar-sweetened beverage)	
2928	Coffee, instant, sweetened, cappuccino flavour, powder, water added
2929	Coffee, instant, sweetened, French flavour, powder, water added
2930	Coffee, instant, sweetened, mocha flavour, powder, water added
502439	Coffee, instant, pre-sweetened, no whitener, powder, water added
502440	Coffee and cocoa (mocha), instant, with whitener, pre-sweetened, powder, water added
502441	Coffee, regular, pre-sweetened with sugar, pre-lightened
504363	Coffee, mocha, with whipped cream
504386	Blended coffee beverage, regular coffee, sweetened, with whipped cream
504722	Blended coffee beverage, decaffeinated coffee, sweetened
504729	Blended coffee beverage, regular coffee, sweetened
504847	Coffee, mocha, without whipped cream
505183	Blended coffee beverage, decaffeinated coffee, sweetened, with whipped cream
Sugar-sweetened tea (beverage type: sugary drink, sugar-sweetened beverage)	
2914	Tea, instant, sweetened, lemon flavour, powder
2915	Tea, instant, sweetened, lemon flavour, powder, water added
4908	Tea, iced, lemon flavour, ready-to-drink
5291	Tea, instant, sweetened, lemon flavour, powder, decaffeinated
6703	Tea, chai latte, prepared with whole milk
502452	Tea, NS as to type, pre-sweetened with sugar
502453	Tea, NS as to type, sweetened, NS as to sweetener (lemon-flavoured)
502454	Tea, NS as to type, sweetened, NS as to sweetener, decaffeinated
502456	Tea, made from powdered instant, pre-sweetened with sugar (NS as to sweetener, iced tea)
504730	Tea, chai latte
Sugar-sweetened hot chocolate (beverage type: sugary drink, sugar-sweetened beverage)	
500028	Hot chocolate, made from dry mix, water added
504779	Hot chocolate, made from dry mix, milk added
Sugar-sweetened yogurt beverage (beverage type: sugary drink, sugar-sweetened beverage)	
6993	Yogourt beverage, fruit flavoured
6994	Yogourt beverage, vanilla flavoured
7119	Yogourt beverage, fruit flavoured, with added vitamin D
7120	Yogourt beverage, vanilla flavoured, with added vitamin D
Regular sports drink (beverage type: sugary drink, sugar-sweetened beverage)	
5962	Sports drink, fruit flavour, ready-to-drink
5963	Sports drink, lemon-lime flavour, ready-to-drink
Regular flavoured water (beverage type: sugary drink, sugar-sweetened beverage)	
7185	Vitamin water, all flavours, sweetened
7187	Vitamin water, lemon/orange flavours, sweetened
7189	Vitamin water, tropical citrus flavour, sweetened, with caffeine
7237	Vitamin water, flavours not lemon/orange, sweetened
Regular energy drink (beverage type: sugary drink, sugar-sweetened beverage)	
7173	Energy drink, coffee flavours
7175	Energy drink, various flavours
7176	Energy drink, coffee flavours, light
7178	Energy drink, with fruit juice
7179	Energy drink, caffeine free
7180	Energy drink, tea flavoured

**Table 4:** (supporting documentation for Fig. 1). Sex-specific prevalence (%) of sugary drink consumption by beverage type and socioeconomic position in children/adolescents on a given day

		Sugary drinks		Sugar-sweetened beverages		100% Juice	
	*N*	Prevalence	Prevalence difference	Prevalence	Prevalence difference	Prevalence	Prevalence difference
		(95% CI)	(95% CI)	(95% CI)	(95% CI)	(95% CI)	(95% CI)
**Females (2–18 years)**							
Overall population	3050	72 (68, 75)		52 (49, 56)		37 (33, 40)	
Highest household education							
Less than high school	94	87 (71, 102)	21 (5, 37)	74 (55, 94)	27 (7, 47)	26 (10, 42)	− 10 (− 26, 6)
High school diploma	471	76 (69, 83)	11 (2, 19)	56 (47, 65)	9 (− 2, 19)	40 (29, 52)	5 (− 7, 17)
Credential below bachelor’s degree	1205	77 (71, 82)	11 (4, 19)	55 (50, 61)	8 (0, 16)	37 (32, 42)	2 (− 4, 8)
Bachelor’s degree or above	1280	66 (61, 70)	Ref	47 (42, 53)	Ref	36 (31, 40)	Ref
Household food security status							
Food insecure	379	77 (70, 84)	6 (− 2, 14)	57 (48, 66)	5 (− 5, 15)	38 (29, 46)	1 (− 7, 10)
Food secure	2671	71 (68, 75)	Ref	52 (48, 55)	Ref	36 (33, 40)	Ref
Household income quintile							
Q1, lowest	598	77 (71, 83)	5 (− 5, 15)	53 (45, 60)	1 (− 12, 13)	42 (34, 49)	7 (− 5, 18)
Q2	577	69 (61, 78)	− 2 (− 15, 10)	49 (42, 56)	− 3 (− 16, 9)	36 (29, 43)	1 (− 8, 10)
Q3	683	72 (65, 78)	0 (− 10, 10)	54 (47, 60)	1 (− 10, 13)	36 (29, 43)	1 (− 9, 12)
Q4	629	69 (62, 76)	− 3 (− 12, 6)	54 (47, 62)	2 (− 9, 13)	33 (26, 41)	− 1 (− 12, 9)
Q5, highest	563	72 (64, 79)	Ref	52 (43, 61)	Ref	35 (27, 42)	Ref
**Males (2–18 years)**							
Overall population	3064	78 (75, 80)		56 (53, 59)		42 (38, 45)	
Highest household education							
Less than high school	96	76 (55, 97)	0 (− 21, 22)	58 (35, 81)	6 (− 17, 28)	39 (18, 61)	− 4 (− 26, 18)
High school diploma	467	80 (72, 89)	4 (− 8, 17)	63 (53, 72)	10 (− 3, 23)	39 (31, 47)	− 4 (− 14, 6)
Credential below bachelor’s degree	1250	79 (75, 82)	3 (− 3, 9)	57 (53, 61)	5 (− 3, 12)	41 (37, 46)	− 2 (− 10, 6)
Bachelor’s degree or above	1251	76 (70, 81)	Ref	52 (47, 58)	Ref	43 (37, 49)	Ref
Household food security status							
Food insecure	394	74 (65, 84)	− 4 (− 15, 8)	59 (48, 69)	3 (− 9, 15)	34 (26, 42)	− 9 (− 18, 0)
Food secure	2670	78 (75, 81)	Ref	56 (52, 59)	Ref	43 (39, 47)	Ref
Household income quintile							
Q1, lowest	593	77 (71, 83)	1 (− 8, 10)	60 (53, 66)	5 (− 6, 15)	39 (32, 46)	1 (− 10, 12)
Q2	581	77 (70, 83)	0 (− 8, 9)	53 (46, 60)	− 2 (− 13, 8)	41 (32, 49)	3 (− 8, 13)
Q3	719	82 (77, 86)	5 (− 2, 12)	58 (51, 65)	3 (− 8, 14)	46 (38, 53)	7 (− 2, 17)
Q4	636	75 (69, 81)	− 2 (− 10, 6)	53 (46, 60)	− 3 (− 13, 8)	43 (37, 50)	5 (− 5, 15)
Q5, highest	535	76 (71, 82)	Ref	55 (47, 63)	Ref	38 (30, 46)	Ref

Data source: 2015 Canadian Community Health Survey–Nutrition. Children/adolescents include respondents aged 2–18 years (females: 3050; males: 3064). SEP indicators include highest household educational attainment, household food security status and household income quintiles. Prevalence difference (PD) represents the absolute difference in mean prevalence and associated 95% confidence intervals (CI) across SEP (education, food security, income quintile). '*N*' represents the number of respondents across sex/age/and SEP indicators.

**Table 5:** (supporting documentation for Fig. 2). Sex-specific prevalence (%) of sugary drink consumption by beverage type and socioeconomic position in adults on a given day

		Sugary drinks		Sugar-sweetened beverages		100% Juice	
	*N*	Prevalence	Prevalence difference	Prevalence	Prevalence difference	Prevalence	Prevalence difference
		(95% CI)	(95% CI)	(95% CI)	(95% CI)	(95% CI)	(95% CI)
**Females (≥ 19 years)**							
Overall population	7219	50 (47, 52)		35 (33, 38)		22 (20, 24)	
Highest household education							
Less than high school	888	48 (41, 54)	− 2 (− 9, 5)	28 (21, 36)	− 6 (− 12, 0)	26 (19, 33)	2 (− 5, 9)
High school diploma	1462	52 (46, 59)	3 (− 3, 8)	40 (35, 44)	5 (− 1, 11)	17 (10, 24)	− 7 (− 15, 1)
Credential below bachelor’s degree	2614	49 (45, 52)	− 1 (− 7, 5)	36 (32, 39)	1 (− 4, 6)	21 (18, 24)	− 3 (− 7, 1)
Bachelor’s degree or above	2255	50 (46, 54)	Ref	34 (30, 39)	Ref	24 (21, 27)	Ref
Household food security status							
Food insecure	742	59 (51, 66)	10 (1, 19)	48 (38, 59)	14 (2, 27)	19 (13, 25)	− 3 (− 9, 3)
Food secure	6477	49 (46, 52)	Ref	34 (31, 37)	Ref	22 (20, 24)	Ref
Household income quintile							
Q1, lowest	1719	53 (49, 57)	11 (1, 20)	38 (33, 43)	10 (− 1, 21)	24 (20, 29)	5 (− 1, 10)
Q2	1603	48 (43, 53)	5 (− 3, 13)	34 (29, 39)	6 (− 5, 17)	19 (16, 23)	0 (− 8, 7)
Q3	1495	52 (46, 57)	9 (− 2, 20)	37 (32, 41)	8 (− 6, 23)	23 (19, 27)	3 (− 3, 9)
Q4	1213	53 (48, 58)	10 (2, 19)	39 (34, 45)	11 (− 4, 26)	22 (15, 29)	2 (− 9, 13)
Q5, highest	1189	42 (34, 51)	Ref	28 (16, 40)	Ref	20 (15, 25)	Ref
**Males (≥ 19 years)**							
Overall population	6409	58 (55, 60)		44 (41, 46)		25 (23, 27)	
Highest household education							
Less than high school	670	52 (45, 59)	− 4 (− 12, 3)	38 (29, 48)	− 1 (− 10, 7)	21 (13, 29)	− 6 (− 16, 3)
High school diploma	1268	58 (52, 64)	2 (− 7, 11)	47 (42, 52)	7 (0, 15)	22 (17, 27)	− 6 (− 12, 1)
Credential below bachelor’s degree	2360	60 (54, 66)	3 (− 6, 12)	47 (41, 53)	8 (− 2, 17)	24 (20, 29)	− 3 (− 9, 3)
Bachelor’s degree or above	2111	56 (52, 61)	Ref	40 (35, 44)	Ref	27 (24, 31)	Ref
Household food security status							
Food insecure	541	60 (47, 72)	2 (− 12, 16)	51 (42, 60)	9 (− 1, 19)	17 (9, 24)	− 9 (− 18, 0)
Food secure	5868	57 (55, 60)	Ref	43 (40, 45)	Ref	26 (23, 28)	Ref
Household income quintile							
Q1, lowest	1125	59 (54, 64)	2 (− 5, 10)	42 (35, 49)	− 2 (− 18, 14)	25 (17, 33)	− 2 (− 11, 7)
Q2	1283	59 (54, 64)	2 (− 7, 10)	47 (42, 52)	4 (− 7, 14)	23 (19, 28)	− 4 (− 10, 3)
Q3	1390	57 (52, 61)	− 1 (− 8, 7)	41 (37, 46)	− 2 (− 13, 8)	26 (22, 29)	− 1 (− 7, 4)
Q4	1200	56 (50, 62)	− 1 (− 12, 10)	44 (38, 49)	0 (− 13, 13)	24 (19, 29)	− 3 (− 10, 3)
Q5, highest	1411	57 (50, 64)	Ref	44 (33, 54)	Ref	27 (23, 31)	Ref

Data source: 2015 Canadian Community Health Survey–Nutrition. Adults include respondents aged 19 years and older (females: 7219; males: 6409). SEP indicators include highest household educational attainment, household food security status and household income quintiles. Prevalence difference (PD) represents the absolute difference in mean prevalence and associated 95% confidence intervals (CI) across SEP (education, food security, income quintile). '*N*' represents the number of respondents across sex/age/and SEP indicators.

**Table 6:** Sex-specific prevalence ratios of sugary drink consumption by beverage type and socioeconomic position in children/adolescents on a given day

		Sugary drinks	Sugar-sweetened beverages	100% Juice
	*N*	Prevalence ratio	Prevalence ratio	Prevalence ratio
		(95% CI)	(95% CI)	(95% CI)
**Females (2–18 years)**				
Highest household education				
Less than high school	94	1.32 (1.09, 1.59)	1.57 (1.18, 2.08)	0.73 (0.39, 1.35)
High school diploma	471	1.16 (1.04, 1.30)	1.18 (0.97, 1.44)	1.14 (0.83, 1.55)
Credential below bachelor’s degree	1205	1.17 (1.06, 1.30)	1.17 (1.00, 1.37)	1.05 (0.88, 1.25)
Bachelor’s degree or above	1280	Ref	Ref	Ref
Household food security status				
Food insecure	379	1.08 (0.98, 1.20)	1.10 (0.92, 1.31)	1.04 (0.82, 1.31)
Food secure	2671	Ref	Ref	Ref
Household income quintile				
Q1, lowest	598	1.07 (0.94, 1.23)	1.01 (0.79, 1.29)	1.20 (0.89, 1.62)
Q2	577	0.97 (0.81, 1.15)	0.93 (0.73, 1.20)	1.04 (0.80, 1.34)
Q3	683	1.00 (0.87, 1.14)	1.03 (0.83, 1.27)	1.04 (0.78, 1.39)
Q4	629	0.96 (0.84, 1.09)	1.04 (0.85, 1.28)	0.96 (0.70, 1.32)
Q5, highest	563	Ref	Ref	Ref
**Males (2–18 years)**				
Highest household education				
Less than high school	96	1.01 (0.76, 1.34)	1.11 (0.75, 1.64)	0.91 (0.52, 1.59)
High school diploma	467	1.06 (0.90, 1.23)	1.19 (0.96, 1.48)	0.91 (0.71, 1.15)
Credential below bachelor’s degree	1250	1.04 (0.96, 1.12)	1.09 (0.95, 1.24)	0.95 (0.79, 1.14)
Bachelor’s degree or above	1251	Ref	Ref	Ref
Household food security status				
Food insecure	394	0.95 (0.82, 1.11)	1.05 (0.86, 1.29)	0.79 (0.61, 1.02)
Food secure	2670	Ref	Ref	Ref
Household income quintile				
Q1, lowest	593	1.01 (0.90, 1.13)	1.09 (0.90, 1.31)	1.03 (0.78, 1.36)
Q2	581	1.00 (0.90, 1.12)	0.96 (0.79, 1.17)	1.07 (0.82, 1.40)
Q3	719	1.07 (0.97, 1.17)	1.05 (0.87, 1.27)	1.19 (0.94, 1.50)
Q4	636	0.98 (0.88, 1.09)	0.95 (0.79, 1.15)	1.13 (0.88, 1.45)
Q5, highest	535	Ref	Ref	Ref

Data source: 2015 Canadian Community Health Survey–Nutrition. Children/adolescents include respondents aged 2–18 years (females: 3050; males: 3064). SEP indicators include highest household educational attainment, household food security status and household income quintiles. Prevalence ratio (PR) represents relative prevalence of consuming on a given day and associated 95% confidence intervals (CI) across SEP (education, food security, income quintile). '*N*' represents the number of respondents across sex/age/and SEP indicators.

**Table 7:** Sex-specific prevalence ratios of sugary drink consumption by beverage type and socioeconomic position in adults on a given day

		Sugary drinks	Sugar-sweetened beverages	100% Juice
	*N*	Prevalence ratio	Prevalence ratio	Prevalence ratio
		(95% CI)	(95% CI)	(95% CI)
**Females (≥ 19 years)**				
Highest household education				
Less than high school	888	0.96 (0.83, 1.10)	0.82 (0.66, 1.02)	1.09 (0.82, 1.44)
High school diploma	1462	1.05 (0.94, 1.18)	1.15 (0.98, 1.36)	0.72 (0.46, 1.13)
Credential below bachelor’s degree	2614	0.97 (0.86, 1.10)	1.03 (0.88, 1.20)	0.88 (0.73, 1.04)
Bachelor’s degree or above	2255	Ref	Ref	Ref
Household food security status				
Food insecure	742	1.21 (1.03, 1.41)	1.42 (1.07, 1.88)	0.85 (0.62, 1.18)
Food secure	6477	Ref	Ref	Ref
Household income quintile				
Q1, lowest	1719	1.25 (1.00, 1.56)	1.35 (0.90, 2.01)	1.23 (0.95, 1.60)
Q2	1603	1.13 (0.94, 1.36)	1.21 (0.81, 1.79)	0.98 (0.68, 1.40)
Q3	1495	1.22 (0.95, 1.56)	1.30 (0.80, 2.12)	1.15 (0.87, 1.53)
Q4	1213	1.24 (1.03, 1.50)	1.39 (0.84, 2.30)	1.09 (0.65, 1.83)
Q5, highest	1189	Ref	Ref	Ref
**Males (≥ 19 years)**				
Highest household education				
Less than high school	670	0.92 (0.79, 1.07)	0.96 (0.77, 1.21)	0.78 (0.51, 1.19)
High school diploma	1268	1.03 (0.88, 1.20)	1.18 (1.00, 1.41)	0.80 (0.61, 1.05)
Credential below bachelor’s degree	2360	1.05 (0.90, 1.24)	1.19 (0.97, 1.47)	0.89 (0.7, 1.12)
Bachelor’s degree or above	2111	Ref	Ref	Ref
Household food security status				
Food insecure	541	1.04 (0.82, 1.32)	1.20 (0.98, 1.46)	0.64 (0.38, 1.08)
Food secure	5868	Ref	Ref	Ref
Household income quintile				
Q1, lowest	1125	1.04 (0.91, 1.19)	0.96 (0.66, 1.40)	0.93 (0.64, 1.34)
Q2	1283	1.03 (0.89, 1.19)	1.08 (0.85, 1.38)	0.86 (0.67, 1.12)
Q3	1390	0.99 (0.87, 1.13)	0.94 (0.74, 1.21)	0.95 (0.76, 1.18)
Q4	1200	0.98 (0.81, 1.19)	1.00 (0.74, 1.36)	0.88 (0.67, 1.15)
Q5, highest	1411	Ref	Ref	Ref

Data source: 2015 Canadian Community Health Survey–Nutrition. Adults include respondents aged 19 years and older (females: 7219; males: 6409). SEP indicators include highest household educational attainment, household food security status and household income quintiles. Prevalence ratio (PR) represents relative prevalence of consuming on a given day and associated 95% confidence intervals (CI) across SEP (education, food security, income quintile). '*N*' represents the number of respondents across sex/age/and SEP indicators.

## Appendix 2. Accounting for energy misreporting in dietary assessments

We included energy misreporting as a sensitivity analysis to account for confounding related to systematic error in self-reported dietary assessments. Energy misreporting was measured based on the ratio of energy intake (EI) to total estimated energy requirement (EER). Children under 12 years were classified as under-reporters (EI:EER < 74%); over-reporters (EI:EER > 135%) and plausible reporters (74% ≤ EI:EER ≤ 135%) (Garriguet, 2009; Jessri et al., 2016a; Jessri et al., 2016b; McCrory et al., 2002; National Academy Press, 2002). Individuals 12 years and over were classified using appropriate cutoff ratios for this age group: under-reporters (EI:EER < 70%), plausible reporters (70% ≤ EI:EER ≤ 142%); over-reporters (EI:EER > 142%). Estimated energy requirements were determined using the National Academies of Sciences, Engineering and Medicine (NASEM) factorial equations, fully described elsewhere (Nomaguchi et al., 2017). The NASEM equations incorporate an individual’s age, sex, physical activity level and body mass index (BMI) (National Academy Press, 2002). For individuals aged 2–17, BMI classification was based on sex- and age-specific cutoff points defined by the WHO (Health Canada, 2017). BMI was calculated using measured height and weight where possible, but when not available, was estimated using validated equations to correct for self-reported height and weight (National Academy Press, 2002; Shields et al., 2011). For individuals with missing BMI, USDA EER equations were applied based on respondent’s age, sex and reported physical activity (Brouillard et al., 2019; National Academy Press, 2002). We grouped respondents with missing information to estimate misreporting, including underweight respondents who are not included in estimating equations, as ‘unclassified’. Results from the misreporting adjusted analyses were consistent with the primary unadjusted results and are available upon request.

## Code availability

Code is available through accessing a public repository found here: https://github.com/BtsmithPHO/Sugary-drinks-descriptive
